# Mucoadhesive PLGA Nanospheres and Nanocapsules for Lactoferrin Controlled Ocular Delivery

**DOI:** 10.3390/pharmaceutics14040799

**Published:** 2022-04-06

**Authors:** Rubén Varela-Fernández, Xurxo García-Otero, Victoria Díaz-Tomé, Uxía Regueiro, Maite López-López, Miguel González-Barcia, María Isabel Lema, Francisco Javier Otero-Espinar

**Affiliations:** 1 Department of Pharmacology, Pharmacy and Pharmaceutical Technology, University of Santiago de Compostela (USC), Campus Vida, 15782 Santiago de Compostela, Spain; rubenvf1@gmail.com (R.V.-F.); xurxo.garcia.otero@gmail.com (X.G.-O.); victoriadiaztome@gmail.com (V.D.-T.); 2 Clinical Neurosciences Group, University Clinical Hospital, Health Research Institute of Santiago de Compostela (IDIS), 15706 Santiago de Compostela, Spain; uxia.regueiro@usc.es (U.R.); maite.lopez.lopez@rai.usc.es (M.L.-L.); 3 Molecular Imaging Group, University Clinical Hospital, Health Research Institute of Santiago de Compostela (IDIS), 15706 Santiago de Compostela, Spain; 4 Clinical Pharmacology Group, University Clinical Hospital, Health Research Institute of Santiago de Compostela (IDIS), 15706 Santiago de Compostela, Spain; miguel.gonzalez.barcia@sergas.es; 5 Department of Surgery and Medical-Surgical Specialties, Ophthalmology Area, University of Santiago de Compostela (USC), Campus Vida, 15706 Santiago de Compostela, Spain; 6 Institute of Materials Imatus, University of Santiago de Compostela (USC), Campus Vida, 15782 Santiago de Compostela, Spain; 7 Paraquasil Group, University Clinical Hospital, Health Research Institute of Santiago de Compostela (IDIS), 15706 Santiago de Compostela, Spain

**Keywords:** nanoparticles, PLGA, lactoferrin, topical ophthalmic administration, nanoprecipitation, protein nanocarriers, keratoconus, corneal ecstatic disorder

## Abstract

Background: the present work describes the preparation, characterization and optimization of eight types of PLGA-based nanosystems (nanospheres and nanocapsules) as innovative mucoadhesive drug delivery systems of lactoferrin, in order to achieve a preclinical consistent base as an alternative pharmacological treatment to different ocular syndromes and diseases. Methods: All different nanoparticles were prepared via two modified nanoprecipitation techniques, using a three-component mixture of drug/polymer/surfactant (Lf/PLGA/Poloxamer), as a way to overcome the inherent limitations of conventional PLGA NPs. These modified polymeric nanocarriers, intended for topical ophthalmic administration, were subjected to in vitro characterization, surface modification and in vitro and in vivo assessments. Results: An appropriate size range, uniform size distribution and negative ζ potential values were obtained for all types of formulations. Lactoferrin could be effectively included into all types of nanoparticles with appropriate encapsulation efficiency and loading capacity values. A greater, extended, and controlled delivery of Lf from the polymeric matrix was observed through the in vitro release studies. No instability or cytotoxicity was proved for all the formulations by means of organotypic models. Additionally, mucoadhesive in vitro and in vivo experiments show a significant increase in the residence time of the nanoparticles in the eye surface. Conclusions: all types of prepared PLGA nanoparticles might be a potential alternative for the topical ophthalmic administration of lactoferrin.

## 1. Introduction

The eye is an airtight organ and presents a high resistance to the absorption of xenobiotics [1,2]. In the past few decades, different strategies have been deeply studied to improve ocular drug retention, ameliorate topical ophthalmic therapeutic effects and minimize the limitations of conventional drug-delivery systems (DDS). At present, several drug-loaded colloidal systems, intended for ophthalmic applications, are reported to provide controlled drug release, as well as better retention in the eye surface as opposed to well-known typical eye drops [3].

Biodegradable polymers, such as poly (lactic-co-glycolic) acid (PLGA), are broadly used as novel DDS due to their compliance with the desirable requirements intended for topical ophthalmic administration (safety, biodegradability, biocompatibility, non-toxicity, sustained drug release and drug site-specific targeting) [4]. PLGA is composed of lactic acid and glycolic acid, providing a large range of physicochemical properties depending on the lactic/glycolic acid ratio.

PLGA nanocarriers may be generally prepared by using different techniques, where nanoprecipitation is one of the most common. Nanoprecipitation involves the addition of a water-miscible organic solution, where a hydrophobic polymer is dispersed into an aqueous solution, prompting a diffusion process that promotes the polymer precipitation [5]. Homogenous and identical particle size distribution is obtained with this technique, leading to an enhanced colloidal stability [6,7,8], reduced production times and expenditures [9,10], and high production yields [11]. Depending on the preparation process, the nanoparticle structure may differ. The drug may be either entrapped into the matrix or adsorbed on the surface [12,13].

The resultant nanosystems present a controlled drug release based on the polymer degradation rate, as well as the diffusion processes of the encapsulated drug [14]. During design and development procedure, the selection of the most appropriate type of PLGA is the main key factor to achieve an effective drug delivery [15]. The lack of mucoadhesiveness hinders its use in topical ophthalmic administration, although PLGA nanoparticles reached good results as DDS [16].

Lactoferrin (Lf) is an iron-binding single-chain glycoprotein composed of two lobes to which Fe^3+^ ions may strongly bind. Lf is acknowledged as a first-line defense protein with a pleotropic functional pattern in humans, including anti-inflammatory [17,18], antibacterial [19,20,21], antiviral [22,23,24] and antitumoral [25,26] actions, among others. Unlike other iron-binding proteins, Lf was observed to promote iron retention in inflamed tissue, as well as stimulate corneal epithelial wound healing [27,28], thereby restricting free radical production and avoiding oxidative damage and inflammation. As consequence of the functions of this glycoprotein, Lf has been proposed for the treatment of different surface ocular pathologies [29], such as (I) dry eye, including Sjögren syndrome and idiopathic dry eye, (II) keratoconjunctivitis, (III) giant papillary conjunctivitis, (IV) irritative and vernal conjunctivitis or (V) viral and bacterial ocular infections, among others.

Additionally, Lf has been identified as a candidate to inhibit hypoxia-inducible factors (HIFs), whose main objective is based on the expression regulation of various genes, including VEGF and, consequently, they are considered as a target for treatment of neovascular ocular diseases such as age-related macular degeneration (AMD), diabetic retinopathy and diabetic macular edema [30].

Likewise, Lf has also been recognized as a key molecule in the pathogenesis of Keratoconus (KC). KC is a progressive and bilateral degenerative ecstatic disorder, characterized by a disorder distinguished by stromal thinning, corneal steepening and irregular astigmatism, which lead to great visual acuity and impaired social health and life quality that affects young adults causing, worsening over time [31]. KC affects all ethnicities and both genders in the same way, whose initial prevalence (1/2000) has significantly increased in the last few years due to new diagnosis techniques [32]. The etiology of KC remains unclear, where environmental, genetic, and behavioral factors act as the main pathophysiological contributors [33,34], not existing approved treatment to prevent or decrease its progression [35], only aimed to slow down its progress [36].

Furthermore, numerous research have recognized the role of oxidative stress and lipid peroxidation pathways in the KC pathogenesis [37,38,39]. Under physiological conditions, the generation of reactive oxygen species (ROS) during metabolism is counteracted by the action of antioxidant enzymes, particularly catalase, superoxide dismutase and glutathione peroxidase, among others. Thus, any alteration in this balance would promote the generation of an oxidative stress phenomenon [40]. Likewise, increased oxidative stress is also associated with several types of non-apoptotic cell death, one of them being ferroptosis (an iron-dependent cell death process). Ferroptosis inducted by the malfunction of the glutathione-dependent antioxidant shield, resulting in the accumulation of lipid peroxidation products [41]. Nevertheless, lipophilic antioxidants s (e.g., α-tocopherol, butylated hydroxytoluene or β-carotene) and iron chelators have proven efficacy as strong suppressors in the ferroptosis cell death prevention [42,43].

In recent years, different works were published aiming to improve the Lf ocular bioavailability due to its great potential in the treatment of several ophthalmic diseases [44]. This improvement may be achieved by increasing the permanence of Lf on the ocular surface or improving Lf corneal permeation. In the present work, Lf-loaded PLGA nanoparticles were prepared by two different modified nanoprecipitation techniques, using a three-component mixture of drug/polymer/surfactant (Lf/PLGA/Poloxamer), as a way to overcome the inherent limitations of conventional PLGA NPs (high initial burst, incomplete release and instability of the encapsulated proteins, among others). These modified polymeric nanocarriers, intended for topical ophthalmic administration, were subjected to in vitro characterization, surface-modification, and in vitro and in vivo assessments.

## 2. Materials

Resomer^®^ RG 502 (Mw: 10,000 Da; lactide:glycolide = 50:50), Resomer^®^ RG 502H (Mw: 17,000 Da; lactide:glycolide = 50:50), Resomer^®^ RG 503 (Mw: 24,000 Da; lactide:glycolide = 50:50) and Resomer^®^ RG 503 H (Mw: 38,000 Da; lactide:glycolide = 50:50) were purchased from Evonik (Essen, Germany). Hydrochloric acid (HCl) and sodium hydroxide (NaOH) and were acquired from Merck (Darmstadt, Germany). Lactoferrin, Polyvinyl Alcohol (PVA) and Pluronic^®^ F68 were acquired from Sigma-Aldrich (St Louis, MI, USA). Visking dialysis tubing cellulose membrane (14,000 g/mol molecular weight cutoff) was purchased from Sigma-Aldrich (St. Louis, MI, USA). All other chemicals and reagents were of the highest purity grade commercially available.

## 3. Methods

### 3.1. Preparation of PLGA Nanoparticles

#### 3.1.1. One-Step Nanoprecipitation Method: Nanospheres

**Figure 1:**
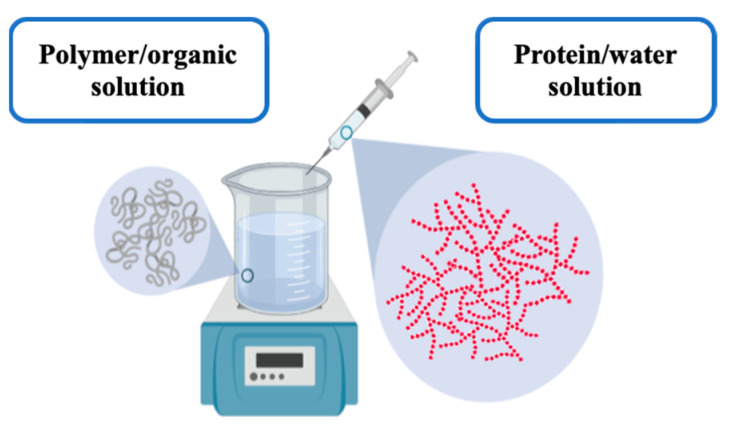
Simplified representation of the PLGA-based nanospheres elaboration process.

The preparation of PLGA nanospheres (NSs) was performed by a modified nanoprecipitation method, based on the elaboration procedure previously developed by Bilati et al. [45], with minor modifications. The polymer was firstly dissolved in DMSO (v = 4 mL). This solution was injected by a syringe/needle system, introducing the needle into the dispersing solution, an 0.5% (*w*/*v*) Pluronic^®^ F68 aqueous solution (v = 40 mL), under magnetic stirring (up to 750 rpm) at room temperature for 30 min. A 1:10 organic:aqueous phase ratio was established for the nanoparticles preparation. Nanoprecipitation immediately occurred when the organic solution was in contact with the aqueous phase. Resulting suspensions were centrifuged 3 times at 4 °C and 50,000 rpm for 1-h cycles and washed with double-distilled water to eliminate the residual DMSO, gradually replacing it with water for subsequent freeze-drying, using trehalose as cryoprotectant (0.5% *w*/*v*). Lactoferrin-loaded PLGA NSs were prepared following the same procedure used for blank nanoparticles, although adding the protein to the aqueous solution before the addition of the organic solution and the subsequently nanoparticle’s formation (see Figure 1). A 1:10 (*w*/*w*) protein:polymer ratio was established for the drug-loaded nanospheres preparation.

#### 3.1.2. Two-Step Nanoprecipitation Method: Nanocapsules

The preparation of PLGA nanocapsules (NCs) was performed by a modified two-step nanoprecipitation method, using a method as developed by Weber et al. [46] and subsequently described by Morales-Cruz et al. [47], with negligible changes. Briefly, Lf was dissolved in an aqueous phase (0.01% *w*/*v*) and then solvent-precipitated by adding acetonitrile at a 1:4 (*v*/*v*) ratio. The resultant protein suspension was stirred for 5 min at room temperature.

Furthermore, PLGA was also dissolved in acetonitrile (C_PLGA_ = 5 mg/mL) under magnetic stirring (500 rpm) at 25 ± 2 °C, and subsequently added to the Lf suspension drop wisely. The resulting mixture was finally added into a maturation medium in a 1:10 (*v*/*v*) ratio, composed of a 1% (*w*/*v*) PVA solution, under vigorous stirring (>750 rpm) and room temperature to promote the nanoparticle formation.

**Figure 2:**
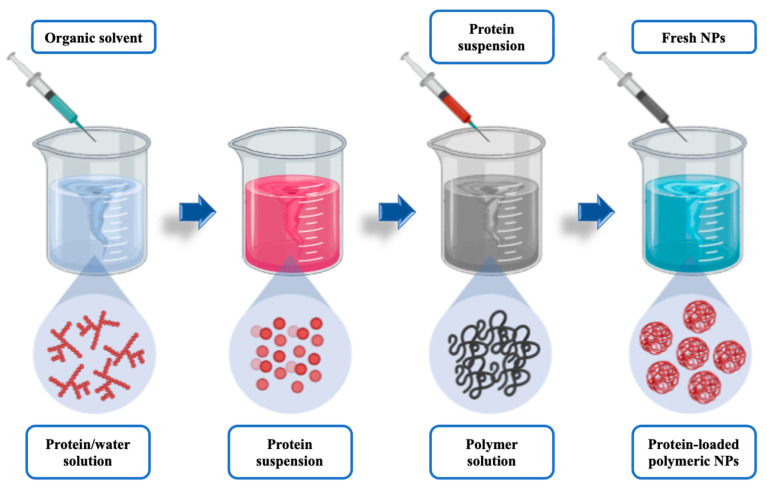
Simplified representation of the PLGA-based nanocapsules elaboration process.

Polymer nanoprecipitation instantaneously occurred upon infusion of the Lf suspension. The resulting lactoferrin-loaded PLGA NCs were the centrifuged for 1 h at 14,000 rpm, the supernatant discarded, and the sediment resuspended in an ocular buffer saline (PBS). A thrice washing step was applied prior to the samples freeze-dried using trehalose as cryoprotectant (0.5% *w*/*v*) (see Figure 2). A 1:10 (*w*/*w*) protein:polymer ratio was established for the drug-loaded nanocapsules preparation.

### 3.2. Physicochemical Characterization of the Nanoparticles

#### 3.2.1. Particle Size, Polydispersity, and Surface Charge

The average particle size, size distribution, and surface potential of the nanoparticles were determined by Dynamic Light Scattering with non-invasive back scattering (DLS-NIBS) at 25 °C. PLGA NPs were 1:10 diluted prior to the analysis. DLS subsets were defined as previously described [44,48]. Each batch was thrice assessed.

#### 3.2.2. Morphological Evaluation

PLGA NSs and NCs were morphologically analyzed two different microscopical techniques, these being: (I) Scanning Electron Microscopy (SEM), and (II) Transmission Electron Microscopy (TEM). An appropriate sample preparation was made prior to the SEM and TEM analysis, as previously described [44,48]. Different batches were used for both types of analysis.

#### 3.2.3. Production Yield (PY)

The PY values of the PLGA NPs were acquired by a centrifugation technique [49], with slight changes. Concisely, predefined volumes of the PLGA NSs or NCs were centrifuged (14,000 rpm, 25 °C, 1 h), the supernatants were discarded, and the sediment was vacuum-dried until constant weight. The PY values were finally estimated as follows (Equation (1)):

PY (%) = (NPs weight)/(Total initial solids weight) × 100(1)

#### 3.2.4. Encapsulation Efficiency (EE) and Loading Capacity (LC)

Encapsulation efficiency and loading capacity of the PLGA NPs were determined by following a centrifugation/spectrophotometry procedure, as previously described [44,48]. EE and LC values were obtained by a centrifugation technique, trailed by the free-drug measurement by UV–Vis spectrophotometry, after polymer precipitation. Briefly, 20 mg of PLGA-based nanoparticles were dissolved into 2.5 mL dichloromethane, followed by a mechanic stirring process (Vortex^®^, VWR International) (Darmstadt, Germany) to promote the polymer dissolution. Later, 5 mL of methanol were appended, and samples were subsequently vortexed for 1 min to foster polymer precipitation. Samples were then centrifuged (5000 rpm, 10 min) and the supernatant was subsequently collected. Unbound Lf was quantified by UV-Vis spectrophotometry at a 280.0 nm wavelength, using a method previously validated by our group. This procedure was repeated using 9 randomized batches for each formulation. The EE (Equation (2)) and LC (Equation (3)) of Lf were respectively calculated as follows:

EE = (Total amount of drug − Amount of unbound drug)/(Total amount of drug) × 100(2)

LC = (Total amount of drug − Amount of unbound drug)/(NPs weight) × 100(3)

### 3.3. Stability Studies

#### 3.3.1. Stability to Storage

ICH guidelines were followed to design the procedure for determining storage stability [50], with negligeable adjustments. PLGA NSs and NCs were freshly prepared, isolated by centrifugation, and resuspended in PBS (pH 7.4, v = 10 mL). Samples (1 mL) were incubated at different temperature environments: (a) 4 ± 2 °C, (b) 25 ± 2 °C, and (c) 37 ± 2 °C for two different periods: 8 h (short-time stability) and 3 months (long-term stability), at 100 rpm and protected from light. Size and size distribution were studied in triplicate for each batch.

#### 3.3.2. Stability to pH

A complete pH interval (2, 4, 6, 7, 8, 10, and 12) was pre-established for the stability study of PLGA NSs and NCs, as previously described [44]. Samples (500 μL) were 1:10 diluted in Milli-Q^®^ water and consequently refrigerated for 24 h. Size, polydispersity, and surface charge were finally analyzed by DLS, as mentioned in previous sections.

#### 3.3.3. Stability to Ionic Strength

A complete ionic strength interval (0.2, 0.4, 0.6, 0.8, 1.2, 1.4, 1.6, and 2 M) was pre-established for the stability study of PLGA NSs and NCs, as previously described [44,48]. Samples (500 μL) were 1:10 diluted in Milli-Q^®^ water and then refrigerated for 24 h. Size, size distribution, and surface charge were finally analyzed by DLS, as mentioned in previous sections.

### 3.4. In Vitro Release Study

The Lf delivery from PLGA NSs and NCs was determined following the dialysis fundamentals previously described as a way to establish the drug diffusion and kinetic behavior from these colloidal systems in a simulated physiological environment. Henceforward, freshly prepared lactoferrin-loaded PLGA NSs and NCs were suspended in PBS (pH 7.4 and ionic strength 0.075 M) for a physiological background proof-of-concept test [51]. The experimental procedure was extensively described in previous works [44,48], where a 1.5 mg/mL NPs concentration ([Lf] = 0.15 mg/mL) was adjusted to fill the donor chamber of the Franz diffusion cells (v = 3 mL). The protein release rate from PLGA NSs and NCs was determined at predetermined times by UV-Vis spectrophotometry at a 280.0 nm wavelength.

### 3.5. Citotoxicity Analysis

#### 3.5.1. Bovine Corneal Opacity and Permeability Test (BCOP)

The BCOP test is an organotypic model commonly employed in the assessment of a great variety of formulations with potential ocular irritancy to fulfill the animal replacement bases.

The BCOP test fundamentals were based on the methodology earlier protocolized by Tchao et al. [52] and improved by Gautheron et al. [53], with minimal adjustments. A standardized procedure for PLGA NPs addition, as well as the permeability and opacity measurements, were established and considerably described in previous works [44]. In this test, corneal opacity changes were estimated by two different experimental techniques (luxmetry and the UV-VIS spectrophotometry), while permeability data was exclusively obtained by UV-Vis spectrophotometry (490.0 nm wavelength). Each formulation was evaluated in triplicate. The resulting permeability and opacity values were used to calculate an in vitro score value (if necessary, see Equation (4)) [53,54]. Furthermore, the classification of PLGA NPs may be performed agreeing to the Kay and Calandra rating [53].

IVIS = mean opacity value + (15 × mean permeability OD_490_ value) (4)

#### 3.5.2. Hen’s Egg Test on the Chorioallantoic Membrane (HET-CAM)

The HET-CAM test has turn into a universal standard test for the assessment of acute eye irritation and corrosion phenomena [55,56], where three potential consequences may be observed in the CAM vasculature and subsequently measured following the Kalweit et al. criteria [57]. The experimental procedure was extensively described in previous works [44]. Briefly, viable eggs were employed after 9 days of incubation under specific environmental conditions [44], using the CAM as the target tissue. Once the formulation was placed onto the CAM, a 5 min inspection period was carried out, taking frames from the beginning to the end of the assay. Results were both individually and combined analyzed. Each formulation was thrice appraised.

### 3.6. Ocular Surface Retention Study

#### 3.6.1. Ex Vivo Corneal Surface Retention

The ex vivo corneal surface retention assay was based on the method firstly defined by Belgamwar et al. and Gradauer et al. [58,59], with minimal adjustments. The experimental procedure was extensively described in previous works [44]. Briefly, freshly excised eyes were placed upwards on specifically designed holders. Fluorescein-stained PLGA NSs and NCs were then added, where the formulation’s excess was collected and reapplied. The residual formulation volume was finally quantified for fluorescence intensity by UV-Vis spectrophotometry at a 490.0 nm wavelength. Each preparation was examined in triplicate. Resulting data was compared with the initial intensity values, and the ex vivo corneal mucoadhesion values were subsequently quantified as follows (Equation (5)):

Mucoadhesion (%) = [((Abs_0_ − V_0_) − (Abs_F_ − V_F_))/(Abs_0_ − V_0_)] × 100 (5)

where Abs_0_ and Abs_F_ indicate the absorbance value for the initial and final fluorescein-stained PLGA NPs, V_0_ denotes the volume instilled to the eyes, and V_F_ designates the final volume of the formulations that was not adsorbed to the mucosa.

#### 3.6.2. In Vivo Corneal Surface Retention Study

##### Evaluation of the Radiolabeling Stability and Efficiency of PLGA-Based Nanoparticles

Radioactive fluoride (^18^F) and gallium (^68^Ga) are two of the most commonly used positron emitters due to their optimal positron features (see details in Table 1), making them suitable for the Positron Emission Tomography (PET) imaging technique. In any case, ^18^F is mostly used in different conjugates, where 2-[^18^F]-fluoro-2-deoxy-D-glucose (^18^F-FDG) and ^18^F-Choline are the most commonly used, while ^68^Ga is mainly used in binary or ternary complexes, such as the ^68^Ga-DOTA (1,4,7,10-tetraazacyclododecane-tetraacetic acid). 

Lately, several methodologies have been investigated to trace a variety of nanosized systems using the aforementioned radiotraces’ labeling [60,61,62,63]. The assessment of PLGA-based NPs radiolabeling efficiency and stability was performed by an incubation procedure, as previously described [44,48]. It must be considered that ^18^F-FDG and ^18^F-Choline were provided by the Cyclotron unity of the University Hospital of Santiago de Compostela (CHUS), while and ^68^Ga-DOTA was provided by the Galician Radiopharmacy Unit. The radiolabeling efficiency of PLGA-based nanoparticles was quantify by the radiotracer activity, both in the supernatant and the remnant vials, considering the incubation interval and the radiotracer decay. Statistical analysis was later employed to acquire the radiolabeling efficiency and labeling stability data.

##### Experimental In Vivo Evaluation of the Ocular Biopermanence of PLGA-Based Nanoparticles

The PET/CT combined technique was used for the in vivo estimation of the PLGA NPs retention time on the ocular surface, as previously described [64]. The in vivo experimental procedure was performed in male Sprague-Dawley rats, whose environmental and feeding conditions were controlled throughout the procedure, according to the ARVO approved guidelines for laboratory animals [65]. The PET/CT acquisition and data processing were extensively described in previous works [44,48]. Each formulation was tested in quadruplicate (two animals, four eyes) to fulfill the laboratory animal regulatory outlines [66].

The PLGA NPs radioactivity versus time diagrams were finally acquired, comparing the resulting data against a control solution (^18^F-Choline buffered solution). A monoexponential decay equation based on a single compartmental model was applied for the remaining formulation versus time fitting by means of the GraphPad Prism^®^ v. 8.41 software (San Diego, CA, USA) and pKSolver^®^, a solver add-in for Excel^®^ (Microsoft, Redmond, WA, USA) [67].

### 3.7. Data Analysis

Pairs of groups were compared by carrying out one-tailed Student’s *t*-test and multiple group comparison was conducted by one-way or two-way analysis of variance, with a 95% significance level (*p* < 0.05), using the GraphPad Prism^®^ v. 8.41 software (San Diego, CA, USA). All data were presented as a mean and standard deviation (mean ± SD). Tukey’s or Bonferroni’s tests were also used for post-hoc contrast.

## 4. Results and Discussion

### 4.1. Preparation of Lactoferrin-Loaded PLGA Nanoparticles

#### 4.1.1. One-Step Nanoprecipitation Method: Lactoferrin-Loaded PLGA Nanospheres

The conventional nanoprecipitation technique is usually based on a solvent displacement phenomenon, where a suitable solvent condition shifted into an inappropriate solvent condition [68]. Nevertheless, the modified one-step nanoprecipitation methodology applied in the present work made it possible to successfully entrap Lf into PLGA nanospheres. Certainly, both protein and polymer were individually dissolved into two different proper solvents. Then, both were changed to a single unsuitable solvent in a one-step mixing process. The obtention of lactoferrin-loaded PLGA NSs was based on the interfacial deposition of the nanoparticle components by a solvent displacement phenomenon between two dissimilar solvents [69].

#### 4.1.2. Two-Step Nanoprecipitation Method: Lactoferrin-Loaded PLGA Nanocapsules

The modified two-step nanoprecipitation method designed for the obtention of lactoferrin-loaded PLGA NCs was successfully applied. This method was based on the assumptions previously described by Giteau et al. [70]. 

The modified two-step nanoprecipitation method previously described (see Section 3.1.2) was based on a double solvent displacement process, by using a desolvating agent (acetonitrile) to promote the protein precipitation and the polymer dissolution. The original methodology was also slightly changed to assure the obtention of nanosized PLGA nanocarriers with high Lf content, avoiding enzyme inactivation or aggregation phenomena.

### 4.2. Physicochemical Characterization of the Nanoparticles

#### 4.2.1. Particle Size Distribution and ζ Potential

Nano-sized particles are known to affect different biological parameters, such as the in vivo tissue distribution, drug content, or stability, among others [71,72,73,74]. 

**Figure 3:**
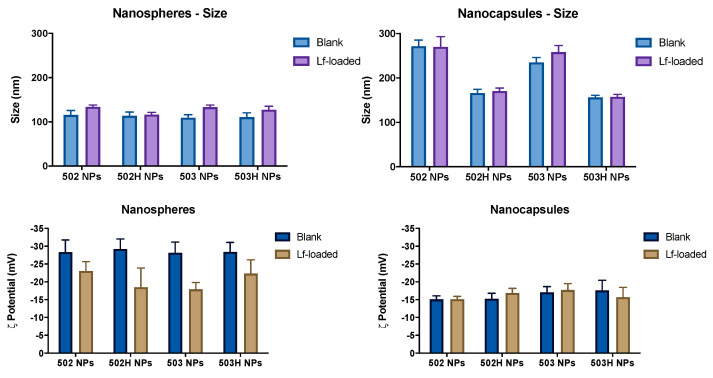
Comparison of size and ζ potential differences for blank and lactoferrin-loaded PLGA-based nanoparticles. A one-way ANOVA statistical analysis was applied, and no statistically significant differences were found among the formulations (*p* > 0.05), regardless of the polymer used.

Figure 3 shows the average size and ζ potential for PLGA NSs and NCs, respectively. The PDI index was minimum for all formulations, suggesting that a homogeneous population of nanoparticles was obtained. Regarding the surface charge, high negatively ζ potential values were obtained, presumably due to the PLGA presence in the surface [75]. This negatively charged net might indicate that these colloidal systems would remain stable over time, which is desirable in order to prevent particle aggregation or coalescence [76].

#### 4.2.2. Effect of Protein Loading on Particle Size and ζ Potential

Figure 3 displays the mean size for blank and lactoferrin-loaded PLGA-based nanoparticles. The resulting data suggest that Lf hardly has any influence on particle size. However, it must be taken into account that the drug:polymer ratio was 1:40, so its impact may be camouflaged by the component proportions in the final formulation. A one-way ANOVA statistical analysis was applied, and no statistically significant differences were found among the formulations (α n.s.), regardless of the polymer used.

On the other hand, surface charge values of Lf-loaded PLGA NSs were lower than blank PLGA NSs (see Figure 3), suggesting that the Lf might not be just encapsulated into the nanocarriers, but also adsorbed to the nanoparticle surface. A one-way ANOVA statistical analysis was carried out, and no statistically significant differences were found among the formulations (α < 0.001), regardless of the polymer used. 

These results are supported by previous studies [77], where the surface potential of the nanoparticles was affected by the molecular distribution of all the compounds and the net formed by the chemical positions. Nevertheless, these ζ potential differences are not significantly observed in the lactoferrin-loaded PLGA NCs (α n.s.) (see Figure 3), supporting the idea that Lf is encapsulated and protected by a polymeric shell, typical of a reservoir colloidal system, without being massively adhered to the polymeric surface.

#### 4.2.3. Morphological Evaluation

SEM and TEM images were scanned both before and after the vacuum-drying process to prove that the physical and morphological characteristics of the PLGA NPs remained unchanged after the dehydration process. Resultant images confirmed the presence of homogenous populations of spherical shape and size below 300 nm. Hence, the vacuum-dried PLGA NPs presumably guarantee the nanoscale size, where size and morphology results were aligned with the morphological evaluation data.

**Figure 4:**
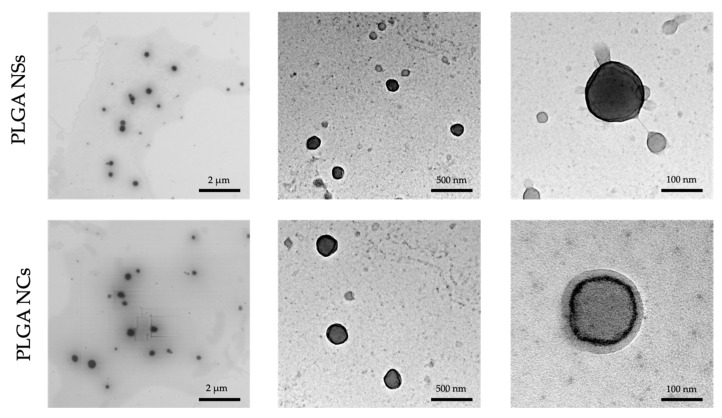
TEM images of lactoferrin-loaded PLGA NSs and lactoferrin-loaded PLGA NCs.

The microstructural analysis by TEM also set that PLGA-based nanoparticles were observed as individual spherical entities, with a homogeneous distribution, spherical in shape and irregular surface particles with well-defined sizes (see Figure 4). These results agree well with previous studies [78,79,80], clearly differentiating the structure of the NSs from the NCs.

#### 4.2.4. Production Yield (PY), Encapsulation Efficiency (EE) and Loading Capacity (LC) of Nanoparticles

**Figure 5:**
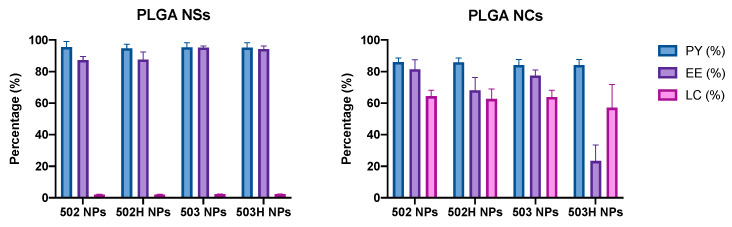
PY, EE, and LC values for PLGA-nanoparticles. No statistically significant differences were found in terms of PY and EE (*p* > 0.05). Nevertheless, considerable differences were observed for LC values, where NCs-based formulations permitted the obtention of higher Lf-embedded content into the polymer matrix, compared to the NSs-based formulations (*p* < 0.0001).

Two different types of PLGA-based NPs were developed for Lf encapsulation. Figure 5 shows the PY, EE and LC of resulting lactoferrin-loaded PLGA-based NPs. The EE determination proved that the nanoprecipitation technique was reproducible and useful, supported by appropriate PY values (above 80%). A one-way ANOVA analysis was performed to assess the existence of dissimilarities among the prepared formulations. The resultant data indicate that no statistically significant differences were found in terms of PY and EE. Nevertheless, considerable differences were observed for LC values, where NCs-based formulations permitted the obtention of high Lf-embedded content into the polymer matrix, compared to the NSs-based formulations, which barely showed any loading capacity (less than 10%). These data are consistent with the nanoparticle preparation method, where NCs show a large amount of drug inside the polymer matrix while, in the NSs, the drug is mostly adhered to the polymeric matrix surface, presumably by electrostatic bonds.

### 4.3. Stability Studies

#### 4.3.1. Stability to Storage

**Figure 6:**
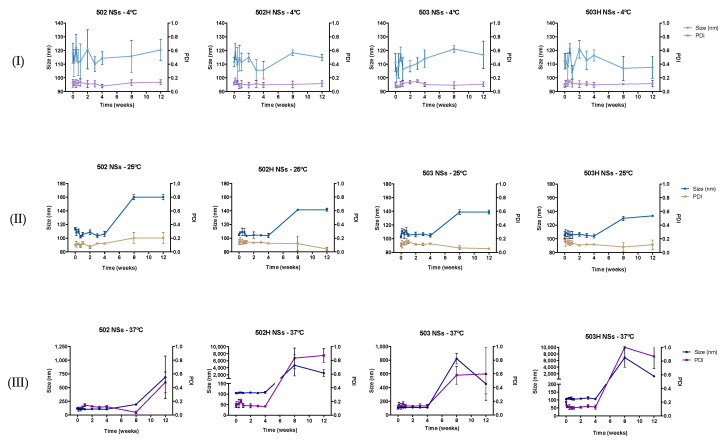
Stability-to-storage study for PLGA-based NSs at three different temperature conditions: (**I**) 4 ± 2 °C, (**II**) 25 ± 2 °C and (**III**) 37 ± 2 °C, respectively.

**Figure 7:**
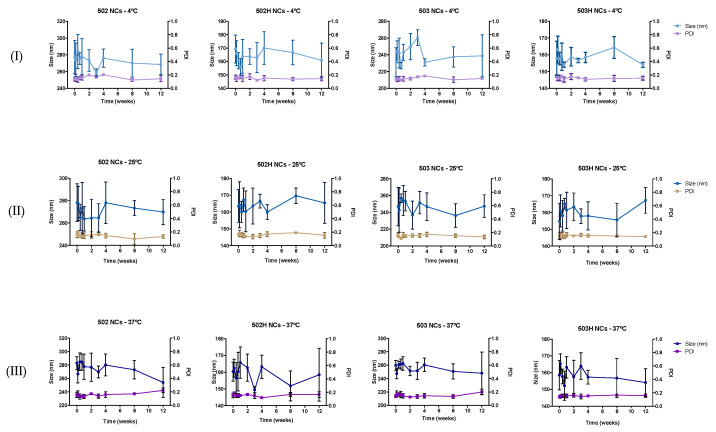
Stability-to-storage study for PLGA-based NCs at three different temperature conditions: (**I**) 4 ± 2 °C, (**II**) 25 ± 2 °C and (**III**) 37 ± 2 °C, respectively.

The resultant data for the long-term stability study proved that both PLGA NPs did not experience any significant modification in their size over a 3-month period (see Figure 6 and Figure 7) for two different temperature sets (4 ± 2 °C and 25 ± 2 °C). Nevertheless, aggregation phenomena were observed in the third temperature set (37 ± 2 °C/60 ± 5% RH) as of the second month. This process may be associated with the glass transition temperature of the PLGA, which varies from 40 to 60 °C (near the studied temperature), leading to an increase in the brittle characteristics of the polymer in physiological-like conditions [81].

#### 4.3.2. Stability to pH

**Figure 8:**
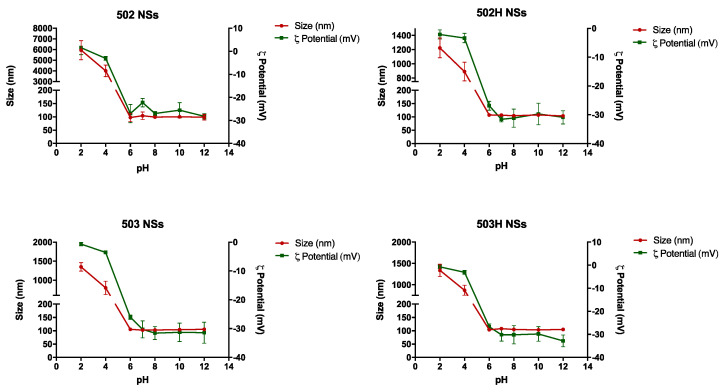
Changes in size and ζ potential values of lactoferrin-loaded PLGA NSs over the studied pH interval.

**Figure 9:**
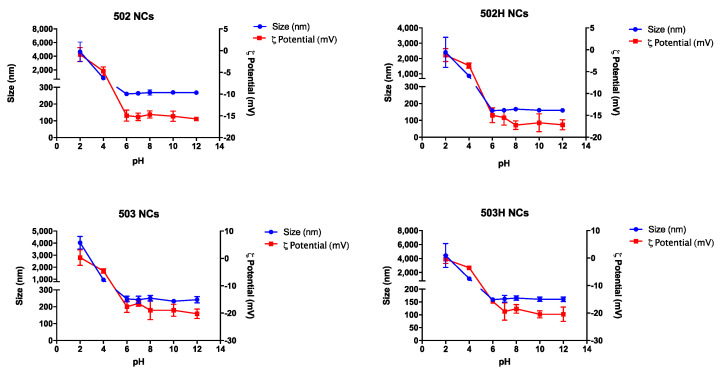
Changes in size and ζ potential values of lactoferrin-loaded PLGA NCs over the studied pH interval.

Collapse, rupture, or aggregation of nanoparticles may appear as a consequence of pH media variations. Figure 8 and Figure 9 show the variations in the physical magnitudes for both PLGA-based nanoparticles along the studied pH interval.

The same pattern was observed along the pH interval in terms of size and ζ potential values for both types of PLGA nanoparticles. These changes are apparently related to the polymeric nature of the resultant nano-sized particles, where PLGA determines the nanoparticle’s ζ potential. Therefore, extremely acidic pH values promoted the nanoparticle’s aggregation due to the interaction between the protonated medium (positive charge) and the carboxyl groups of the polymer (negative charge), where a nullification of the NPs surface charge was observed. Nonetheless, PLGA-based nanoparticles remained stable in in the rest of the pH interval. Hence, PLGA-based colloidal systems would be suitable for topical ophthalmic administration [82].

#### 4.3.3. Stability to Ionic Strength

**Figure 10:**
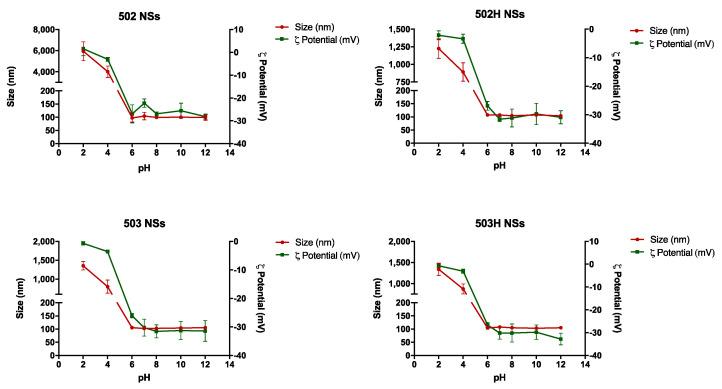
Changes in size and ζ potential values of lactoferrin-loaded PLGA NSs over the studied ionic strength interval.

**Figure 11:**
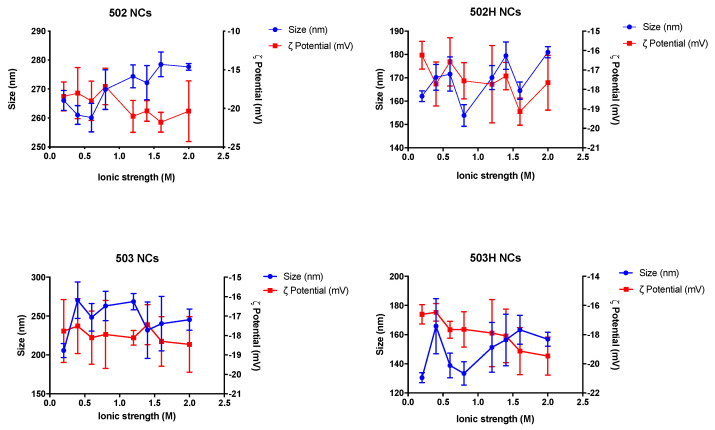
Changes in size and ζ potential values of lactoferrin-loaded PLGA NCs over the studied ionic strength interval.

Collapse, rupture, or aggregation of nanoparticles may appear because of ionic strength media variations. Figure 10 and Figure 11 show the resultant data for both types of Lf-loaded PLGA nanoparticles over the studied ionic strength interval, respectively.

The colloidal steadiness of both types of nanoparticles remained stable in spite of dysphysiological conditions due to variations in the ionic strength values of the medium, where a surface charge rise seemed to be directly related to an increase in the NaCl concentration.

Ionic strength may condition the physical stability of PLGA nanoparticles by promoting possible time-dependent precipitation, sedimentation, or aggregation phenomena [83]. Nonetheless, Lf-loaded PLGA NPs remained stable in terms of changes in size, turbidity, and particle compactness in spite of changes in the ionic strength values of the media over the studied period.

### 4.4. In Vitro Release Study

The two key release mechanisms linked to drug-loaded PLGA nanosystems are usually diffusion and bioerosion/degradation. The release rate is initially controlled by the diffusion mechanism while the bioerosion/degradation processes modulate the final stage of the release period. Preceding studies have also proven the influence of drug release mechanisms and many other factors (porosity, water content, polymer–drug and drug–drug interactions, among others) in drug diffusion and degradation kinetics from PLGA-based DDS [83].

**Figure 12:**
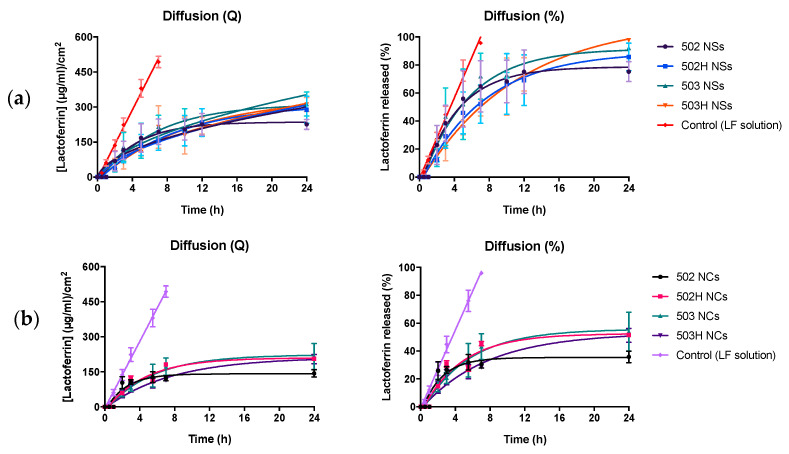
In vitro release study of Lf from PLGA NSs (**a**) and NCs (**b**). The first graph embodies the raw amount of Lf corrected by the available surface released from the PLGA NSs over the 24 h period. The second graph represents the percentage of Lf released from the PLGA NSs over the 24 h period. No statistically significant differences were observed among all the PLGA-based formulations (*p* > 0.05).

The pilot in vitro release study of lactoferrin from the PLGA NPs evidenced that both nanospheres and nanocapsules exhibited a controlled release profile, compared to a Lf aqueous solution (control solution), as observed in Figure 12. The in vitro lactoferrin release profiles allow the elucidation of a biphasic pattern, as follows: (I) a first initial burst release due to the lactoferrin desorption from the particle surface, and (II) an asintotic release phase, resulting from the lactoferrin diffusion from the polymer matrix because of the polymer erosion processes. It must be considered that the polymer’s autocatalytic degradation is based on the excision of ester bonds, resulting in a decrease in the molecular weight, being faster in the matrix center and becoming more pronounced in larger systems [84].

On the other hand, a lower molecular weight leads to less hydrophobia and therefore a higher water absorption capability, increasing their hydrolysis and bioerosion rate. Thus, the release of Lf was expected to be faster in the nanoparticles made with Resomer^®^ RG 502 and Resomer^®^ RG 502H, compared to those made with Resomer^®^ RG 503 and Resomer^®^ RG 503H. Nevertheless, no statistically significant differences were observed among all the formulations.

In addition, the release rate can be also affected by interactions between the drug and the polymer. These interactions and their impact on the subsequent release profiles have been deeply examined in previous studies [81,82,83]. However, no statistically significant differences were noticed among all the formulations despite differences in the hydrophobicity of the polymers, possibly due to the similarity on the lactic and glycolic acids’ content.

The resulting in vitro release profiles were adjusted to different kinetic models. The best correlation was obtained with the Hopfenberg and Peppas-and-Korsmeyer kinetic models (with *n* values in the range of 0.57–0.93), suggesting that the surface erosion of both NSs and NCs was caused by the dissolution, swelling and polymer chain scission processes [85,86,87], and the protein diffusion was the predominant release mechanism (Table 2). These results were supported by data previously published in other scientific articles [88,89,90].

### 4.5. Citotoxicity Analysis

#### 4.5.1. Bovine Corneal Opacity and Permeability Test (BCOP)

**Figure 13:**
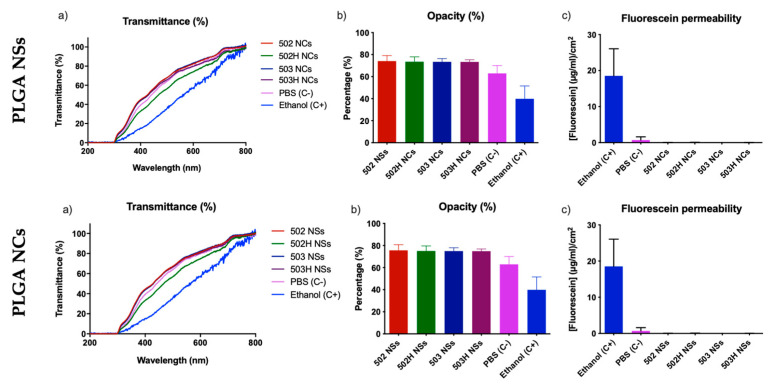
Resulting data of the BCOP test for PLGA-based NSs (**top**) and NCS (**bottom**). (**a**) final corneal transparency values measured by UV-Vis spectrophotometry; (**b**) final corneal transparency values measured by luxmetry, and (**c**) fluorescein permeability measured by UV-Vis spectrophotometry.

The BCOP test is an appropriate ex vivo tissue model that allows the depth of injury assessment by distinguishing the moderate, severe, and extremely severe ocular irritant substances. Figure 13a show the transparency variations measured by UV-Vis spectrophotometry for the lactoferrin-loaded PLGA NSs and NCs. Figure 13b show the transparency variations measured by luxmetry for the lactoferrin-loaded PLGA NSs and NCs, respectively.

All PLGA-based formulations showed an in vitro IS of 0 (IS = 0), exhibiting no cytotoxicity effects in terms of transparency modification, compared to control formulations. These results are also supported by the fluorescein permeability data (see Figure 13c), where no fluorescein passage was observed through the corneas after the administration of the formulations.

#### 4.5.2. Hen’s Egg Test on the Chorioallantoic Membrane (HET-CAM)

The CAM is a no innervated tissue and constitutes a well-developed vascularization model and an easy-to-study alternative strategy for ocular irritation assessment due to the complete inflammatory process response, similar to that induced in the Draize test, as it can be performed with greater efficiency and faster measurements than other in vivo tests [91,92,93]. Thus, the HET-CAM assay was used to assess the cytotoxicity and biocompatibility of the prepared nanoparticles.

**Figure 14:**
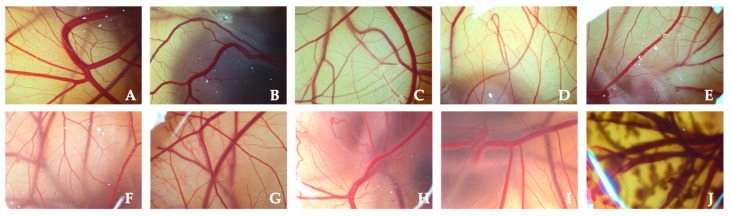
Resulting images of CAM membranes after the PLGA-based nanoparticles administration during the HET-CAM test, compared to the control solutions: (**A**) 502 PLGA NSs; (**B**) 502H PLGA NSs; (**C**) 503 PLGA NSs; (**D**) 503H PLGA NSs; (**E**) 502 PLGA NCs; (**F**) 502H PLGA NCs; (**G**) 503 PLGA NCs; (**H**) 503H PLGA NCs; (**I**) NaCl aqueous solution; and (**J**) NaOH aqueous solution.

PLGA-based NPs showed no cytotoxicity effects (IS = 0) (see Figure 14), comparing them with control formulations (0.9% (*w*/*v*) NaCl and 1.8% (*w*/*v*) NaOH aqueous solutions, used as negative and positive control formulations). These results are in accordance with formerly published studies [94] and agree with the results previously obtained by the BCOP test (see Section 4.5.1) confirming that these nanoparticles are biocompatible and non-toxic.

### 4.6. Ocular Surface Retention Study

#### 4.6.1. Ex Vivo Corneal Surface Model

**Figure 15:**
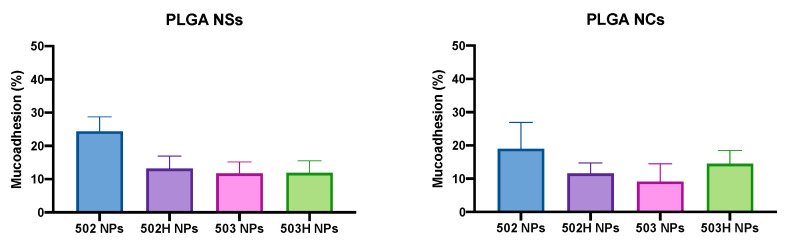
Ex vivo mucoadhesion data for PLGA-based nanoparticles. A one-way ANOVA analysis was applied for the PLGA NSs, showing no differences among the formulations (*p* > 0.05), except for the 502 PLGA NSs, which showed a higher mucoadhesion than the rest, being statistically significant (*p* < 0.02). Nevertheless, the same one-way ANOVA analysis was applied for the PLGA NCs, and no significant differences were observed between the prepared formulations, even for the 502 PLGA NCs compared to the others (*p* > 0.05).

The ex vivo corneal surface bioadhesion determination was adapted from the previous works described by Belgamwar et al. (2009) and Gradauer et al. (2012) [58,59], with minor modifications. Figure 15 displays the mucoadhesion percentage for all the PLGA-based NPs. The resulting data reinforce the possibility of using PLGA-based nanoparticles as an alternative technological strategy for the topical ophthalmic administration of Lf.

A one-way ANOVA analysis was applied for the PLGA NSs, showing no differences among the formulations, except for the 502 PLGA NSs, which showed a higher mucoadhesion than the rest, being statistically significant. Nevertheless, the same one-way ANOVA analysis was applied for the PLGA NCs, and no significant differences were observed between the prepared formulations, even for the 502 PLGA NCs compared to the others.

The PLGA NSs and NCs ex vivo mucoadhesion values are lower than observed in our group for previous studies made with chitosan and sulfobutylether-β-cyclodextrin/chitosan nanoparticles [44] and nanostructured lipid carriers [48], so it seems that PLGA-based nanoparticles have less mucoadhesiveness. This is presumably associated with the surface charge of the nanoparticles, where chitosan-based NPs showed a positive surface charge, which allowed electrostatic interactions with the sialic acid groups of mucin, whereas PLGA nanoparticles showed a negative surface charge, so that their main mechanism of ocular adhesion may be associated with ease of permeation through the corneal layers. Likewise, it must be considered that the ex vivo conditions directly correlate with in vivo conditions, where different factors are involved and influence the pharmaceutical form administration, such as tearing and blinking, among others (see details in Section 4.6.2).

#### 4.6.2. In Vivo Ocular Surface Permanence Study

##### Evaluation of the Radiolabeling Stability and Efficiency of PLGA-Based Nanoparticles

**Figure 16:**
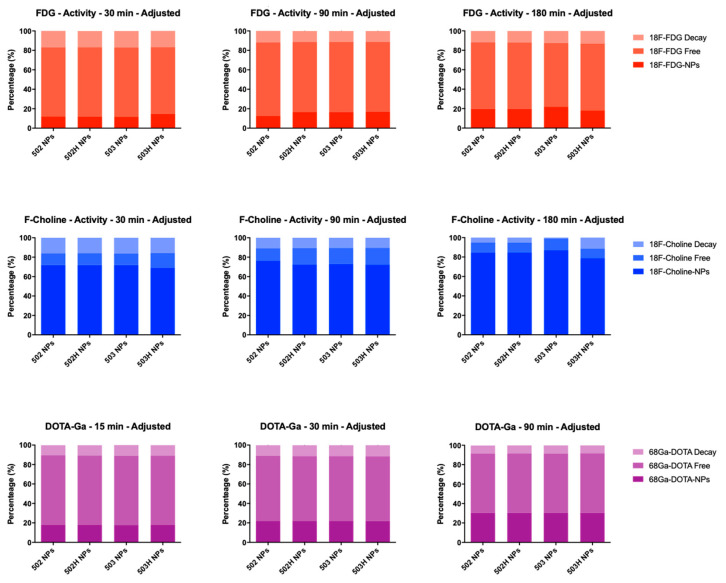
^18^F-Choline (**top**), ^18^F-FDG (**middle**) and ^68^Ga-DOTA (**bottom**) radiolabeling stability and efficiency for PLGA-based nanoparticles over time.

Figure 16 shows the ^18^F-FDG, ^18^F-Choline and ^68^Ga-DOTA radiolabeling stability and efficiency for PLGA-based NPs, respectively. As observed, ^18^F-FDG and ^68^Ga-DOTA led to low radiolabeling efficiency (under 50%), although a radiolabeling stability was observed along the studied interval time, while ^18^F-Choline displayed a great radiolabeling efficiency (over 80%) and stability over the assayed period (up to 3 h), assuring suitable properties for PLGA NPs radiolabeling study. Based on these results, ^18^F-Choline was chosen as the radiotracer for subsequent in vivo radiolabeling studies.

The labeling efficiency of the studied radiotracers are closely related with the radiotracer charge and the ζ potential of the nanoparticles. PLGA nanoparticles, similarly to the nanostructured lipid carriers (NLCs) developed in previous works [48], have a negative surface charge and, therefore, have a maximum radiolabeling efficiency with ^18^F-Choline, which has a positively charged net. On the contrary, chitosan-based nanoparticles [44] that showed a positively charged surface, better radiolabeling performance was obtained with the ^18^F-FDG that has a negatively charged net. In the case of ^68^Ga-DOTA, the radiotracer is also negatively charged, although the radiolabeling efficiency of PLGA NPs was lower, presumably due to the larger size of the radiotracer.

##### Experimental In Vivo Evaluation of Ocular Surface Permanence

**Figure 17:**
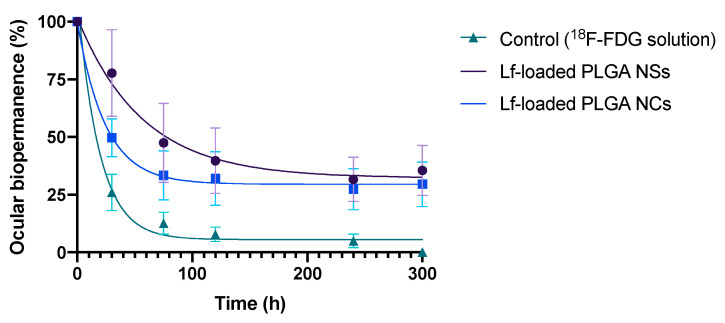
Ocular biopermanence of a ^18^F-Choline radiolabeled PLGA-based nanoparticles and a ^18^F-FDG aqueous solution (control), assessed pondering the primary biopermanence data (%) in the ROI. Statistically significant differences (*p* < 0.05) were observed regarding PLGA-based formulations and 18F-FDG solution.

Figure 17 shows the ocular surface permanence of the ^18^F-Choline-radiolabeled PLGA-based nanoparticles for a 300-min period, compared to the ^18^F-FDG solution, used as control. A ^18^F-FDG solution was used as a standard due to the fact that the ^18^F-Choline is positively charged in its free form, so that it would ionically interact with the negative charge of the sialic acid groups of the mucin layer, resulting in erroneous biodistribution values. On the contrary, ^18^F-FDG, presenting a free negative charge, would not bind to the mucin layer, perfectly simulating the administration of a control substance. For the sake of clarification, an analysis of the ^18^F-Choline-labeled PLGA nanoparticles was carried out to see if this radiotracer modified the size and surface charge, and the results showed that there was no change in either parameter (unpublished data), ensuring that the labeling procedure is suitable for this type of delivery system. The corneal surface retention study of the PLGA-based NPs was measured by the ^18^F-Choline radioactivity assessment in a PET system.

**Figure 18:**
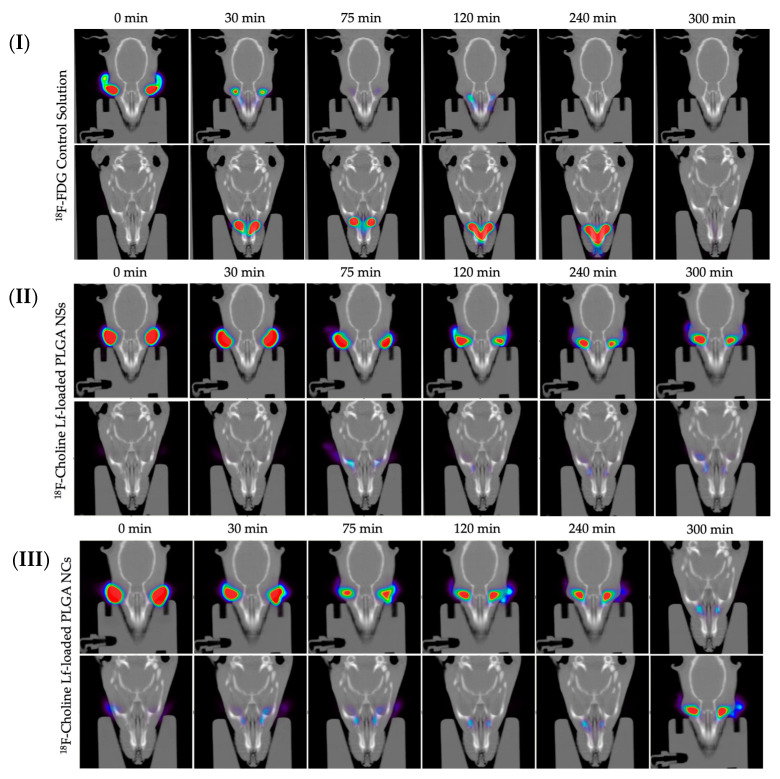
Fused PET/CT images during the 5 h studied interval. (**I**) ^18^F-FDG buffered aqueous solution, (**II**) ^18^F-Choline radiolabeled PLGA NSs and (**III**) ^18^F-Choline radiolabeled PLGA NCs.

The ocular surface permanence of the PLGA-based nanoparticles was carried out on rats using ^18^F-Choline as a radiotracer. A PET equipment was employed in order to trail the radioactivity evaluation over the studied time (Figure 18). In the present work, a longer t_1/2_ was observed for both Lf-loaded PLGA NSs and NCs, compared to the ^18^F-FDG control solution, despite the fact that both formulations presented a tear-like composition [94]. Resultant data were precisely adjusted to a monoexponential decline outline by a single compartmental model (R = 0.9875 for Lf-loaded PLGA NSs, R = 0.9977 for Lf-loaded PLGA NCs, and R = 0.9930 for the 18F-FDG solution) (see Figure 17). The resultant data are in accordance with PET data of previous studies [95], and suggest that PLGA-based nanoparticles showed mucoadhesive, cell uptake and corneal penetration characteristics.

Table 3 shows the pharmacokinetics factors (k, t_1/2_, MRT, AUC, and % dose 30 min) of the ^18^F-Choline radiolabeled PLGA-based nanoparticles and ^18^F-FDG standard solution. Statistically significant differences (*p* < 0.05) were observed regarding PLGA-based formulations and ^18^F-FDG solution for the studied period, where Lf-loaded polymeric nanoparticles exhibited a better ocular retention outline.

The ocular permanence of the PLGA-based nanoparticles obtained by the PET technique followed the same pattern that the mucoadhesive Lf-loaded CS/TPP and CS/SBE-β-CD NPs, as well as the Lf-loaded NLC [44]. Corneally, chitosan-based NPs displayed a t_1/2_ of 114 ± 72 h for CS/TPP NPs, and 60 ± 20 h for CS/SBE-β-CD NPs, respectively, while Lf-loaded NLC showed a t_1/2_ of 107.82 ± 38.10 h. Thus, results confirmed the great bioadhesive capability of the PLGA-based formulations in the ocular mucosa.

## 5. Conclusions

In recent decades, polymeric nanoparticles have risen as promising alternatives for drug delivery in the biomedical field. Indeed, PLGA-based formulations have turned into promising DDS for topical ophthalmic administration, compared to the conventional pharmaceutical forms. This is mainly associated with their numerous advantages, such as their versatility, ease of scale, high stability, biodegradability and biocompatibility, among others. 

The present work reports the design, development and physicochemical characterization of tunable-size Lf-loaded PLGA-based nanoparticles by modified nanoprecipitation methods. Lf-loaded PLGA-based nanoparticles showed appropriate average particle size, size distribution and ζ potential, as well as a spherical and uniform shape. Different drug:polymer ratios were tested in order to obtain the lowest particle size with maximum production yield, encapsulation efficiency, and loading capacity values. Analogously, all formulations showed good stability to storage, pH and ionic strength in the studied ranges. A controlled lactoferrin release was also confirmed, confirming their use as suitable DDS for topical ophthalmic delivery. Resultant data also demonstrated that both nanosystems (NSs and NCs) interact with ocular surface, guaranteeing an optimal contact between the formulation and the corneal mucosa for at least 5 h, with no evidence of tissue cytotoxicity.

In conclusion, different PLGA-based NPs were proposed as biocompatible DDS for the lactoferrin administration via topical ophthalmic route, reinforced by several preclinical studies in order to accomplish a reliable base as a new pharmacological alternative for the keratoconus treatment over the invasive surgical procedures. Despite this, better in vitro/in vivo correlations should be made in order to broaden the knowledge of the potential of these nanosystems as a new approach for the keratoconus treatment, as well as establish its reproducibility with the backdrop of other biological features.

## Figures and Tables

**Table 1:** Physicochemical characteristics of different radiotracers (^18^F and ^68^Ga).

Radiotracer	t_1/2_ (min)	E_β+,max_ (KeV)	β^+^ Intensity (%)
^18^F	109.7	635	97
^68^Ga	67.71	1899	89

**Table 2:** Release data of Lf-loaded PLGA NSs and NCs into the Hopfenberg ($M_{t}/M_{\infty }=1-{\left[kt\right]}^{n}$), Higuchi ($M_{t}/M_{\infty }=k\sqrt{t}$), Peppas and Korsmeyer ($M_{t}/M_{\infty }=kt^{n}$), and monoexponential diffusion kinetics models.

	Hopfenberg			Higuchi		Peppas and Korsmeyer		
Formulation	*k*	*n*	R	*k*	R	*k*	*n*	R
502 NSs	0.0377	3	0.9418	26.04	0.9265	50.57	0.58	0.9326
502H NSs	0.0302	3	0.9679	23.99	0.9363	14.21	0.70	0.9610
503 NSs	0.0394	3	0.9340	27.42	0.9061	20.09	0.92	0.9163
503H NSs	0.0293	3	0.9789	24.00	0.9364	11.12	0.80	0.9787
502 NCs	0.0215	3	0.7839	14.85	0.7924	0.57	0.57	0.7965
502H NCs	0.0243	3	0.8798	17.85	0.8336	0.81	0.81	0.8826
503 NCs	0.0237	3	0.9639	17.82	0.8909	0.93	0.93	0.9722
503H NCs	0.0170	3	0.8914	12.76	0.8756	0.70	0.70	0.9043

**Table 3:** Ocular biopermanence parameters for ^18^F-Choline radiolabeled PLGA-based nanoparticles and ^18^F-FDG control (named as “control” in the table) formulations.

Formulation	K (min^−1^)		t_1/2_ (min)		% Dose 30 min	
	Mean	SD	Mean	SD	Mean	SD
PLGA NSs	0.008	0.0092	93.31	37.71	77.73	18.78
PLGA NCs	0.014	0.0107	51.32	17.45	49.72	8.19
^18^F-FDG control	0.044	0.012	16.27	3.81	23.31	5.89
^18^F-Choline control	0.013	0.012	53.19	11.28	42.90	5.33

## Data Availability

Not applicable.
